# PAX3 expression patterns in ocular surface melanocytes

**DOI:** 10.1038/s41598-025-90318-3

**Published:** 2025-04-11

**Authors:** Eva Ulrich, Sebastian Kistenmacher, Gottfried Martin, Ursula Schlötzer-Schrehardt, Berthold Seitz, Claudia Auw-Hädrich, Günther Schlunck, Thomas Reinhard, Naresh Polisetti

**Affiliations:** 1 Eye Center, Medical Center - Faculty of Medicine, University of Freiburg, Killianstrasse 5, 79106 Freiburg, Germany; 2 Department of Ophthalmology, University of Erlangen-Nürnberg, Erlangen, Germany; 3 Department of Ophthalmology, Saarland University Medical Center, Homburg, Saar Germany

**Keywords:** Limbal stem cells, Limbal stem cell niche, Limbal niche cells, Mesenchymal stromal stem cells, Melanocytes, Limbal epithelial progenitor cells, Melanoma, Limbal melanoma, Conjunctival melanoma, PAX3, PAX6, Aniridia, Limbal stem cell deficiency, Diseases, Eye diseases, Corneal diseases, Stem cells, Stem-cell niche

## Abstract

PAX3, a transcription factor essential for neural crest development and melanocyte progenitors, is expressed in various melanocytic tissues. However, its role in ocular surface tissues remains poorly understood. This study investigated the expression patterns of PAX3 in the limbal stem cell niche, specifically in limbal epithelial progenitor cells (LEPC), limbal melanocytes (LM), and limbal mesenchymal stem cells (LMSC). Additionally, PAX3 expression was studied in conjunctival/limbal melanoma specimens. Immunohistochemical analysis revealed predominant PAX3 expression in LM as well in the conjunctival melanocytes, suggesting distinct roles in stem cell regulation and melanocyte maintenance. Notably, PAX3 was significantly upregulated in conjunctival/limbal melanoma tissues compared to healthy counterparts, with expression co-localizing with melanocyte markers (Melan-A, HMB45, SOX10) and the proliferation marker Ki-67 in melanoma cells. These findings suggests that while PAX3 expression is restricted to melanocytes in limbal/conjunctival tissues and its dysregulation may play a crucial role in conjunctival/limbal melanoma development. Further investigation into mechanisms by which PAX3 influences corneal pathophysiology and contributes to conjunctival/limbal melanoma pathogenesis could identify potential therapeutic targets for this aggressive ocular malignancy.

**Supplementary Information:**

The online version contains supplementary material available at 10.1038/s41598-025-90318-3.

## Introduction

The limbal stem cell niche is a specialized anatomic region of the ocular surface that harbors a population of limbal epithelial stem/progenitor cells (LEPC) responsible for regulating corneal epithelial homeostasis and facilitating tissue repair mechanisms^1^. This specialized niche allows for intricate interactions between LEPC and various supporting cells, including limbal melanocytes (LM) and limbal mesenchymal stromal cells (LMSC)^2^. LM, located in the basal epithelial layer of the corneoscleral limbus, are each associated with about 10 LEPC, forming clusters resembling the melanin units in the skin^3–6^. Their primary function is to transfer melanin granules to neighboring LEPC to protect them from UV damage and the degree of pigmentation correlates with the state of LEPC differentiation, with the most highly pigmented populations harboring the most immature progenitor cells^7^. Additionally, LM have been shown to support the growth and maintenance of LEPC in tissue-engineered corneal epithelial constructs and exhibit remarkably potent anti-inflammatory, immunomodulatory, and anti-angiogenic properties, rendering them attractive candidates for therapeutic applications^8,9^. However, understanding the molecular characteristics of LM, which have been addressed only in a few studies, is critical for advancing our knowledge of corneal epithelial regeneration and maintenance.

The highly conserved PAX (paired box) family of transcription factors plays a crucial role in embryogenesis and is implicated in various developmental disorders and tumorigenesis^10–12^. PAX6, in particular, controls many downstream genes in eye morphogenesis and corneal homeostasis^13,14^. Haploinsufficiency of PAX6 proteins leads to aniridia and limbal stem cell deficiency and eventually visual impairment^15^. PAX6 expression has been documented in human LM and its loss has been associated with altered melanogenesis in aniridia-associated keratopathy^11^. In contrast, PAX6 expression was absent in the vimentin^+^ cells in the limbal epithelial basal layers, suggesting either Langerhans cells or melanocytes^16^. Consistent with these findings, our recent study reported the lack of PAX6 expression in adult LM within the limbal stem cell niche^17^. Therefore, investigating the presence and potential roles of other PAX family members, specifically in LM, is warranted. PAX3, a key regulator of neural crest development and melanocyte progenitors^18^, is expressed in melanoma tissues and cell lines^19,20^, normal skin melanocytes, and melanocytic lesions^21,22^, contributing to cell survival and growth of cells of the melanocytic lineage^19,20^. However, the role of PAX3 in ocular surface tissues, particularly in melanocytes, has not been well characterized, and given the importance of LM in supporting the limbal stem cell niche and their potential therapeutic applications, investigating the expression and the function of PAX3 in these cells could provide valuable insights into the regulatory mechanisms governing their behavior and interactions within the niche.

In this study, we aimed to investigate the expression profiles of PAX family in cultured LEPC, LMSC and LM using qRT-PCR to identify the predominant PAX gene family expressed in each cell type. Focusing on the highly expressed PAX3, we further validated its expression in LM through immunocytochemistry and Western blotting. Additionally, we examined the immunohistochemical localization of PAX3 in healthy human corneal-conjunctival tissue sections as well as in conjunctival/limbal melanoma specimens.

## Materials and methods

Human donor corneoscleral tissues (*n* = 6; mean age 75.2 ± 10.9 years; <16 h after death) not suitable for transplantation and organ-cultured corneoscleral tissue (*n* = 3; mean age 60.3 ± 1.4, post-mortem duration 16.1 ± 6.1 h; culture duration 33.3 ± 1.4 d) after retrieval of corneal endothelial transplants, with appropriate research consent provided by the Lions Cornea Bank Baden-Württemberg, were used as described previously^23^. The donor’s or their next of kin’s informed agreement to corneal tissue donation was obtained. Experiments using human tissue samples were approved by the Institutional Review Board of the Medical Faculty of the University of Freiburg (25/20) and adhered to the principles of the Helsinki Declaration.

Conjunctival or conjunctival/limbal melanoma tissues were chosen for characterization studies due to the well-defined origin of these tumors arising from the conjunctiva, whereas the precise anatomical site of origin for limbal melanoma remains uncertain. Conjunctival/limbal melanoma samples (*n* = 6, Supplementary Table 1) from patients who underwent tumor resection at the Eye Center of the University of Freiburg with appropriate consent were used as described earlier^24^ with ethical approval by the Institutional Review Board of the Medical Faculty of the University of Freiburg (481/19). The patient cohort comprised three females and three males, with ages ranging from 46 to 68 yrs. The cohort included six patients with conjunctival or conjunctival/limbal melanoma, comprising three females and three males aged 46 to 68 years. The first case involved a 63-year-old female with a long-standing history of conjunctival pigmentation since 1995, which had progressively enlarged and extended to the limbus. The second case was a 60-year-old female who presented with a vascularized lesion that had increased in size over one year. Clinical suspicion indicated conjunctival intraepithelial neoplasia with overlapping involvement of the limbus. The third patient, a 67-year-old male, had a fleshy, vascularized tumor. Similarly, the fourth case, a 68-year-old male, displayed a pigmented conjunctival tumor. The fifth patient, a 46-year-old male, presented with a prominent, progressively enlarging pigmented tumor at the limbus, with a medical history of schizophrenia. Lastly, the sixth case involved a 56-year-old female with a pigmented conjunctival tumor that had been noted for approximately six months.

### Immunohistochemistry

Immunohistochemistry was carried out on paraffin sections or frozen section as previously described^25^. Briefly, corneoscleral tissue samples (*n* = 6), organ-cultured corneoscleral tissue (*n* = 3), conjunctival/limbal melanoma samples (*n* = 6), were either embedded in optimal cutting temperature (OCT) compound and frozen in liquid nitrogen or embedded in paraffin. Antigen-retrieval was performed for paraffin embedded tissue sections in Tris-EDTA or Citrate buffer at 95 °C using a pressure cooker.

For single/double immunostaining on paraffin sections, immunofluroscence staining was performed. The same dewaxing, rehydration and antigen retrieval procedures described above were followed for immunofluorescence. Sections were blocked with Ultra vision protein block (TA-125-PBQ, Thermo Scientific). The samples were then incubated with primary antibodies (Supplementary Tables 2 & 3) diluted in antibody diluent (DAKO). Alexa-488-,568-,558-conjugated secondary antibodies (Life Technologies, Carlsbad, CA, USA) were used to detect antibody binding. The stained sections were mounted in Vectashield antifade mounting media with DAPI (Vector, Burlingame, CA, USA). Immunolabeled samples were examined using a laser scanning confocal microscope (TCS SP − 8, Leica, Wetzlar, Germany).

Immunofluroscence staining on cryo sections was performed as described previously^9^.

### Cell isolation and culturing

LEPC (CD90^−^CD117^−^), LMSC (CD90^+^CD117^−^), and LM CD90^−^CD117^+^) from cadaveric limbal tissues were isolated and cultured as described previously^26^.

### Western blotting

Western blotting was carried out as previously described^17^. Briefly, RIPA buffer (R0278, Sigma-Aldrich) with a protease inhibitor cocktail (complete Tablets Mini, Roche, Basel, Switzerland) was used to isolate total protein from cells. A colorimetric test (PierceTM BCA Protein Assay Kit, Thermo Fisher Scientific) was used to determine the total protein concentration. SDS-PAGE was used to separate 10 µg of total protein under reducing conditions, and immunoblot analyses were carried out with primary antibodies (Supplementary Table 2) followed by horseradish peroxidase-labeled anti-mouse or rabbit IgG (Jackson ImmunoResearch Europe, Ely, UK). Enhanced chemiluminescence Western blot detection reagent (GE Healthcare, München, Germany) and a FUSION FX imager/fusion software (Vilber Lourmat, Collégien, France) were used to visualize protein bands.

### Real-time RT-PCR

RNA was isolated from cultured cells using the RNeasy Micro Kit (Qiagen, Hilden, Germany) including an on-column DNase digestion step according to the manufacturer’s instructions. First-strand cDNA synthesis was performed using 2 µg of RNA and Superscript II reverse transcriptase (Invitrogen, Karlsruhe, Germany) as previously described^27^. PCR reactions were run in triplicate using TaqMan Probe Mastermix (Roche Diagnostics, Mannheim, Germany), according to the manufacturers’ recommendations. Primer sequences (Sigma-Aldrich) are given in Supplementary Table 4. For normalization of gene expression levels, ratios relative to the housekeeping gene *GAPDH* were calculated by the comparative *C*_T_ method (ΔΔ*C*_T_). Genes were considered as differentially expressed when their expression levels exceeded a two-fold difference in all specimens analyzed (*n* = 5).

### Statistics

All assays were performed in at least four independent experiments. Statistical analyses were performed using the GraphPad Instat statistical package for windows (Version 6.0; Graphpad Software Inc., La Jolla, CA, USA) and all the data were presented as mean ± standard error of the mean (SEM). The statistical significance (p value < 0.05) was determined with Mann–Whitney U test.

## Results

### Expression of PAX gene family in cultured limbal cells

**Fig. 1:**
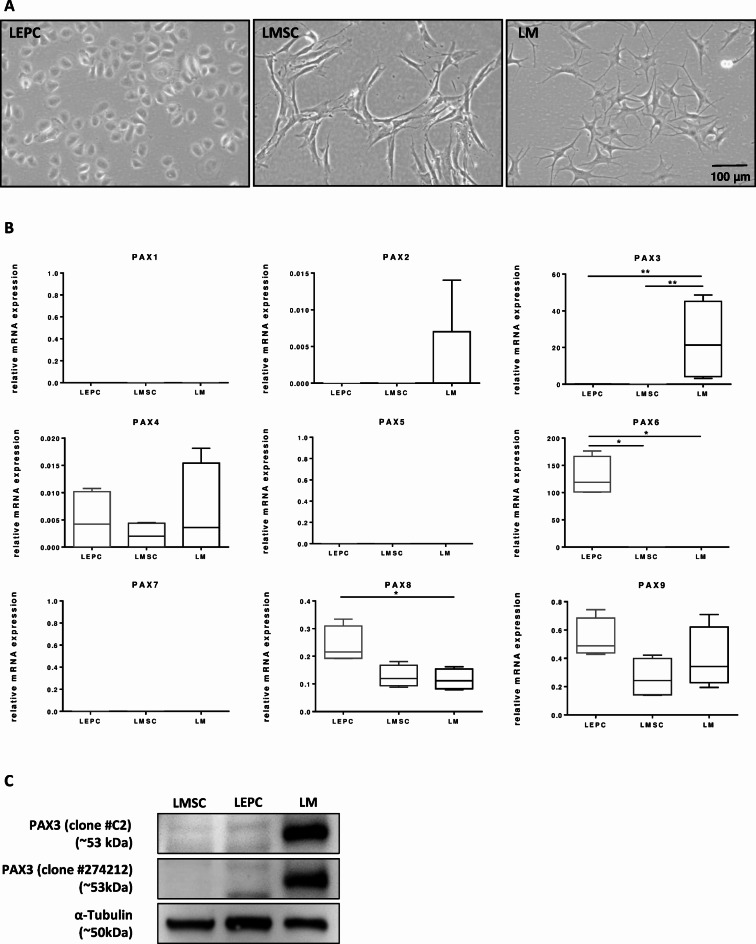
Characterization of limbal cell types and PAX expression. (**A**) Phase contrast images showing the spindle-shaped morphology, elongated with prominent nucleolus of limbal mesenchymal stromal cells (LMSC); large, flattened, smooth bodies with multiple dendrites of limbal melanocytes (LM); small cuboidal epithelial phenotype of limbal epithelial progenitor cells (LEPC). (**B**) mRNA expression levels of PAX gene family in cultured LEPC, LMSC and LM. Data are expressed as means (2^−ΔCT^) ± SEM (*n* = 5); *p < 0.05; **p < 0.01. Mann-Whitney *U* test. (**C**) Western blot analysis of PAX3 expression (using two different clones) in LM, LMSC and LEPC. α-tubulin serves as a loading control. Uncropped versions are available in Suppl. Figure 1C.

Isolated LEPC (CD90^−^CD117^−^), LMSC (CD90^+^CD117^−^), and LM (CD90^−^CD117^+^) had similar cultural and functional characteristics as described previously (phase contrast micrographs, (Fig. 1A))^26^. To analyze the expression patterns of PAX transcription factors in the limbal stem cell niche, initial screening was carried out at the RNA level in the cultured LEPC, LMSC and LM. Primers specific for each PAX family members were designed to exclusively amplify the respective transcripts. Differential gene expression analysis (*n* = 5) revealed that PAX6 was predominantly expressed in LEPC compared to LMSC and LM, while PAX3 showed predominant expression in LM compared to LMSC and LEPC (Fig. 1B). To corroborate the PAX3 expression findings, additional primers targeting distinct regions of the PAX3 gene were employed, yielding concordant results (Supplementary Fig. 1A). The expression of PAX4, PAX8 and PAX9 expression was relatively low (Supplementary Fig. 1B), with no significant differences was among the cell types analyzed except PAX8 (Fig. 1B). The expression of other PAX family members, including *PAX1*,* PAX2* (minimal level in LM*)*,* PAX5* and *PAX7*, were not detected under experimental conditions tested (Fig. 1B).

To validate the mRNA expression of *PAX3* in LM at the protein level, immunocytochemistry and Western blot analysis were carried out on cultured LM, LMSC and LEPC populations. Western blot analysis using two distinct clones of PAX3 antibodies was conducted on total protein extracted from LEPC (P1), LMSC (P2) and LM (P2). The results revealed PAX3 expression (band size ~ 53 kDa) in cultured LM but not in LMSC and LEPC (Fig. 1C). However, non-specific bands of different sizes from the predicted molecular weight were observed in LEPC and LMSC (Suppl. Figure 1C).

**Fig. 2:**
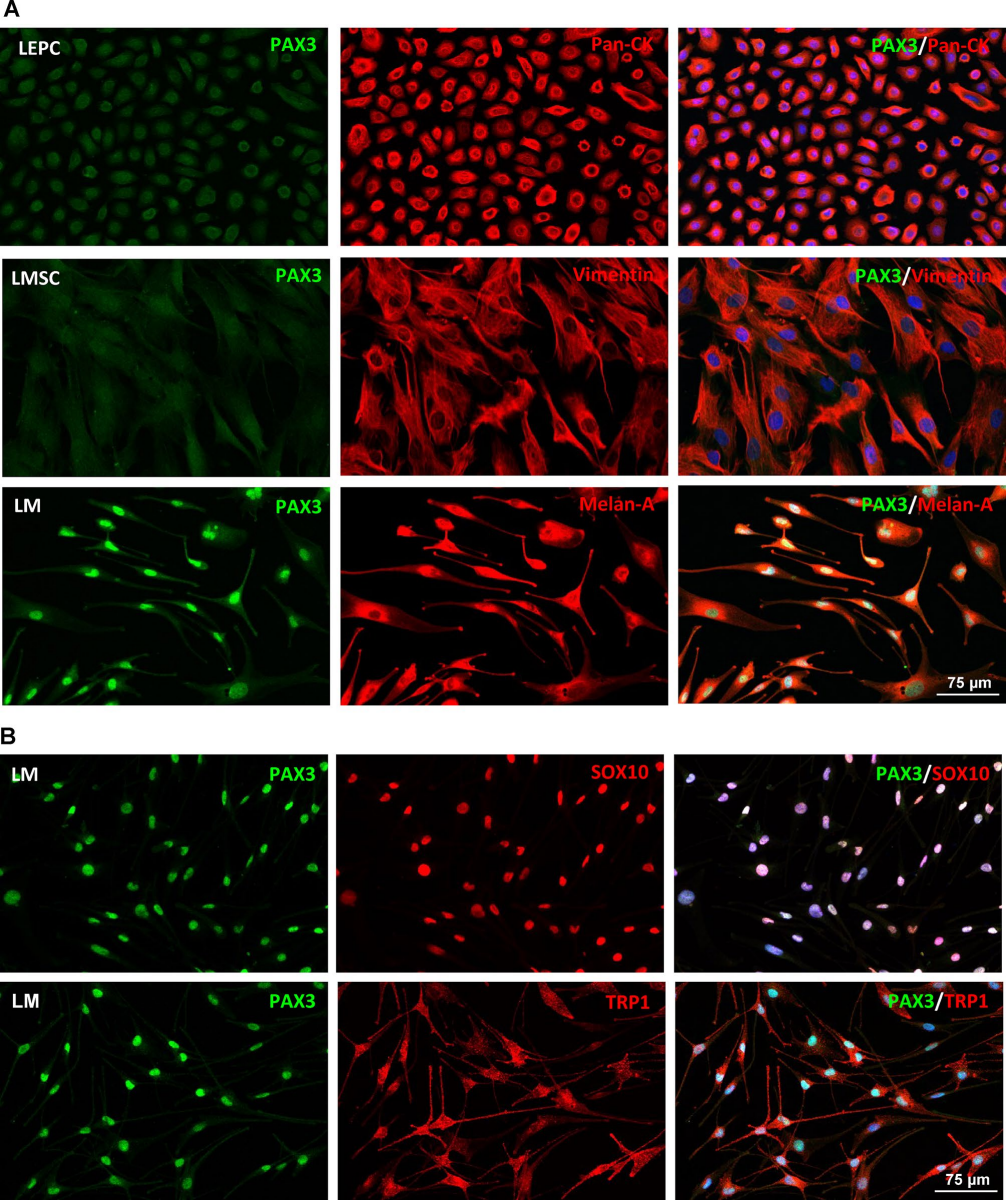
Immunocytochemical analysis of PAX3 expression in limbal cell types. (**A**) Immunocytochemical staining of cultured limbal melanocytes (LM) showing the nuclear PAX3 expression co-stained with Melan-A; limbal mesenchymal stromal cells (LMSC) positive for vimentin but negative for PAX3; and limbal epithelial progenitor cells (LEPC) positive for cytokeratins (pan-CK) but negative for PAX3. (**B**) Double immunostaining of LM revealing co-expression of PAX3 (green) in TRP1^+^ and SOX10^+^ cells (red). Nuclear counterstaining with DAPI (blue).

To further validate protein expression at the cellular level, immunocytochemistry analysis was carried out on cultured LEPC (P1), LMSC (P2) and LM (P2). Nuclear *PAX3* (green) expression was observed in Melan-A positive LM (Fig. 2A), but not in vimentin (red) positive LMSC or keratin (red) positive (pan-CK) LEPC (Fig. 2A). To further confirm these findings, double immunostaining was performed with other melanocytic markers SOX10 and tyrosinase related protein 1 (TRP1). The double immunostaining analysis corroborated the co-expression of PAX3 (green) in TRP1- (red) and SOX10 (red)-positive cells (Fig. 2B).

### Immunohistochemistry

To examine the PAX3 expression at the limbal stem cell niche, immunohistochemistry was performed on non-cultured corneal-conjunctival tissue to examine the native state expression. At the same time, we also checked the expression of PAX3 in conjunctival tissue, which is adjacent to the limbal tissue. Moreover, PAX3 expression was also checked on organ-cultured corneoscleral tissue to see effect of culture conditions. Double immunostaining was performed on tissue sections using epithelial (PAX6, CK12, CK15), stromal (vimentin), and melanocytic (Melan-A and SOX10) markers. The dashed line represents the basement membrane in all the images.

**Fig. 3:**
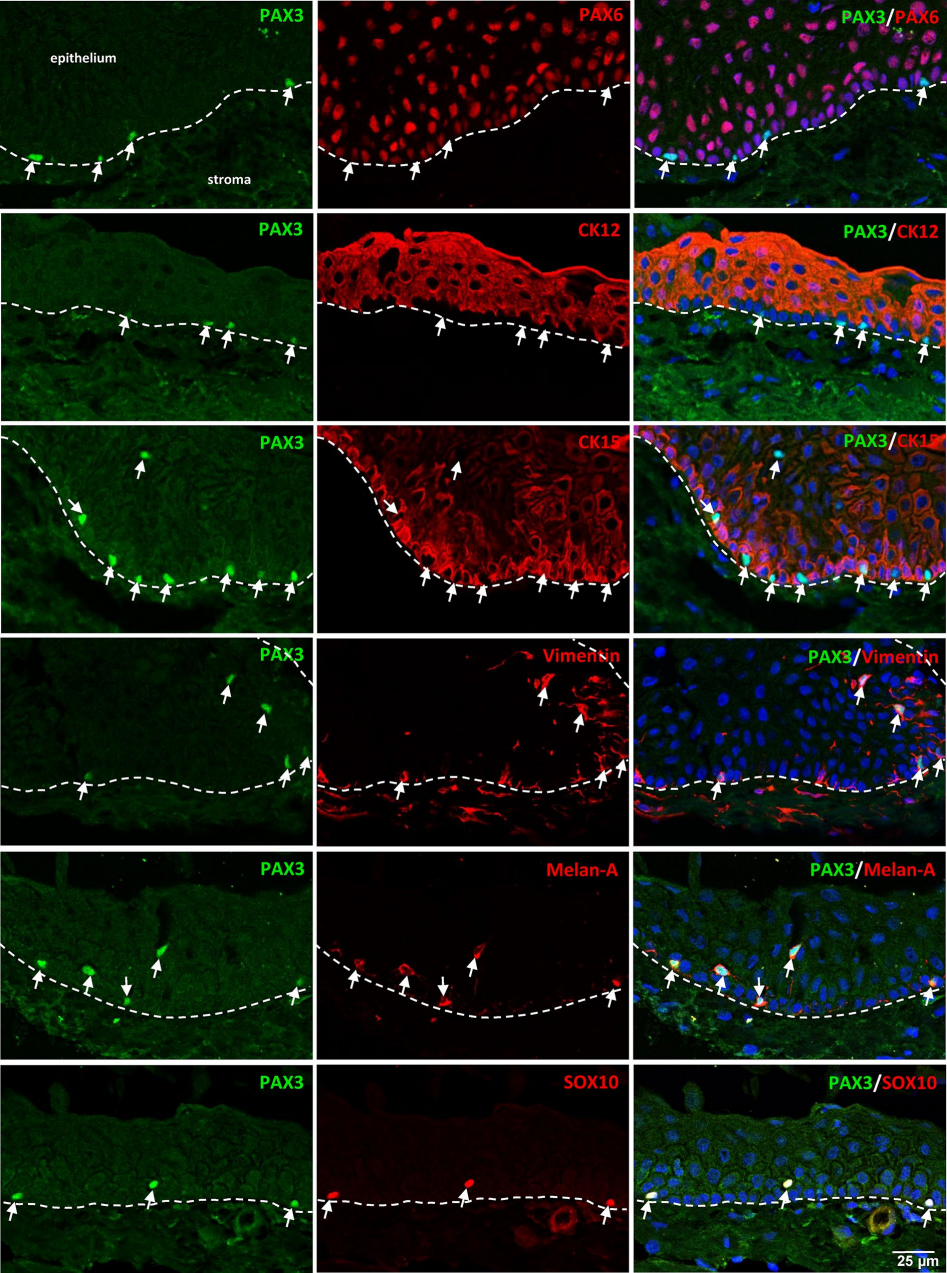
PAX3 expression in limbal tissues. Double immunostaining of corneoscleral sections revealing PAX3^+^ cells (arrows) at the basal layer of limbal epithelium. These cells do not express PAX6, cytokeratin 12 (CK12), or CK15 (red) but do express vimentin (arrow, red) and melanocyte markers Melan-A and SOX10 (red). The dashed line represents the basement membrane. Nuclei are counterstained with DAPI (blue).

Immunofluorescent double staining of limbal tissue sections revealed the presence of PAX3^+^ cells (green, arrows) localized to the basal layers of the limbal epithelium (Fig. 3). Notably, these PAX3^+^ cells did not express markers of ocular surface epithelial cells (PAX6), corneal epithelial cells (CK12), or LEPC (CK15) (red, Fig. 3). Instead, the PAX3^+^ cells exhibited positive staining for the mesenchymal marker vimentin (red, Fig. 3). In contrast, PAX3 expression was not observed in the vimentin^+^ stromal cells of the limbal stromal tissue (red, Fig. 3). Consistent with findings of cell culture studies, the PAX3^+^ cells (arrows, green) in the limbal tissue sections were positive for the melanocyte markers Melan-A (red) and SOX-10 (red, Fig. 3).

**Fig. 4:**
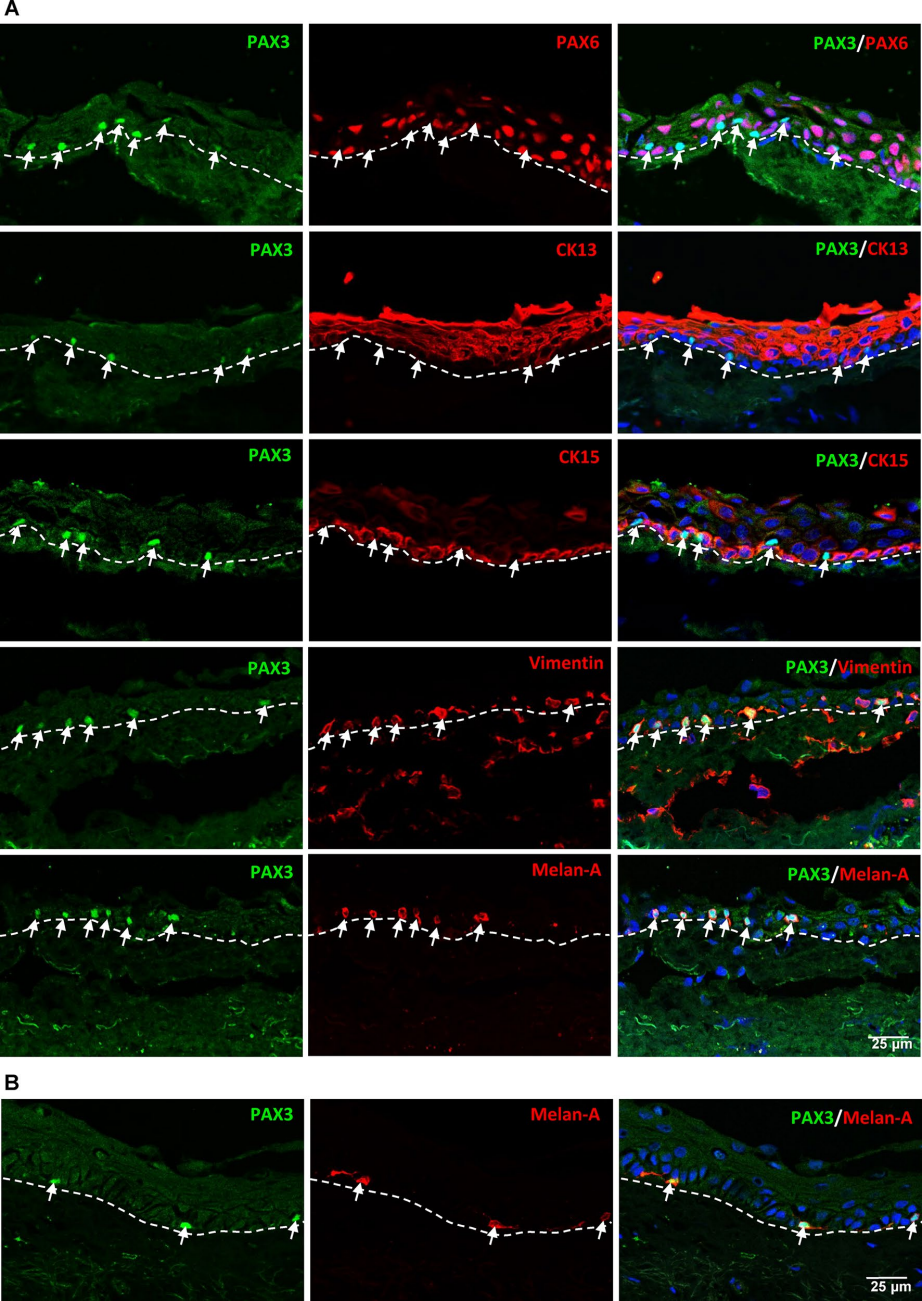
PAX3 expression in conjunctival and organ-cultured corneoscleral tissues. (**A**) Double immunostaining of conjunctival tissue sections showing PAX3^+^ cells (arrows) at the basal layer of conjunctival epithelium. These cells do not express PAX6, cytokeratin 13 (CK13), or CK15 (red) but do express vimentin (arrow, red) and melanocyte markers Melan-A (red) and SOX10. The dashed line represents the basement membrane. (**B**) Double immunostaining of organ-cultured corneoscleral tissue demonstrating PAX3^+^ cells (arrows) expressing Melan-A (red). Nuclear counterstaining with DAPI (blue).

In conjunctival tissue sections, akin to the observations in limbal tissues, PAX3^+^ cells (arrows, green) did not exhibit co-expression of PAX6 (red), the conjunctival epithelial marker CK13 (red), or the epithelial progenitor marker CK15 (red, Fig. 4A). Instead, these PAX3^+^ cells (green, arrows) expressed vimentin (red) as well as the melanocyte markers Melan-A and SOX10 (arrows, red, Fig. 4A). Consistent with limbal tissues, PAX3 expression was not detected in the stromal cells of the conjunctiva (red, vimentin, Fig. 4). Organ-cultured limbal tissue explants displayed a similar pattern to freshly isolated limbal tissues, with PAX3^+^ cells expressing the melanocyte marker Melan-A (arrows, red, Fig. 4B).

### PAX3 expression patterns in melanoma

**Fig. 5:**
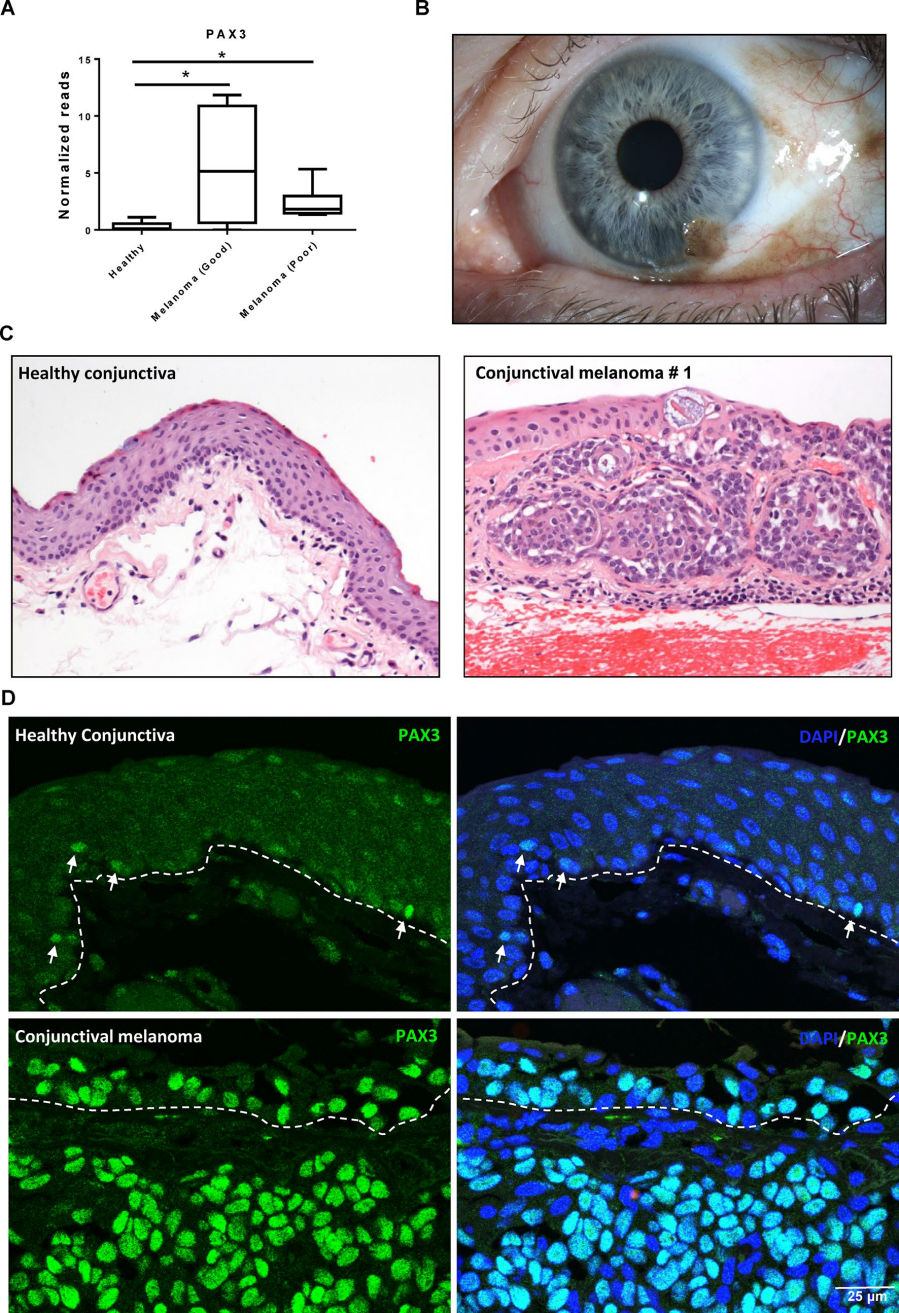
PAX3 expression in conjunctival melanoma. (**A**) RNA-sequencing data analysis showing significant upregulation of PAX3 in conjunctival melanoma (both poor and good prognosis) compared to healthy conjunctival tissue. (**B**) Representative clinical image of conjunctival melanoma: pigmented lesion visible on temporal conjunctiva at 5 o’clock position. (**C**) Representative histological specimens of normal conjunctiva and melanoma. (**D**) Immunofluorescent staining of paraffin sections showing strong expression of PAX3 (green) in melanoma compared to healthy tissue. Nuclei are counterstained with DAPI (blue).

**Fig. 6:**
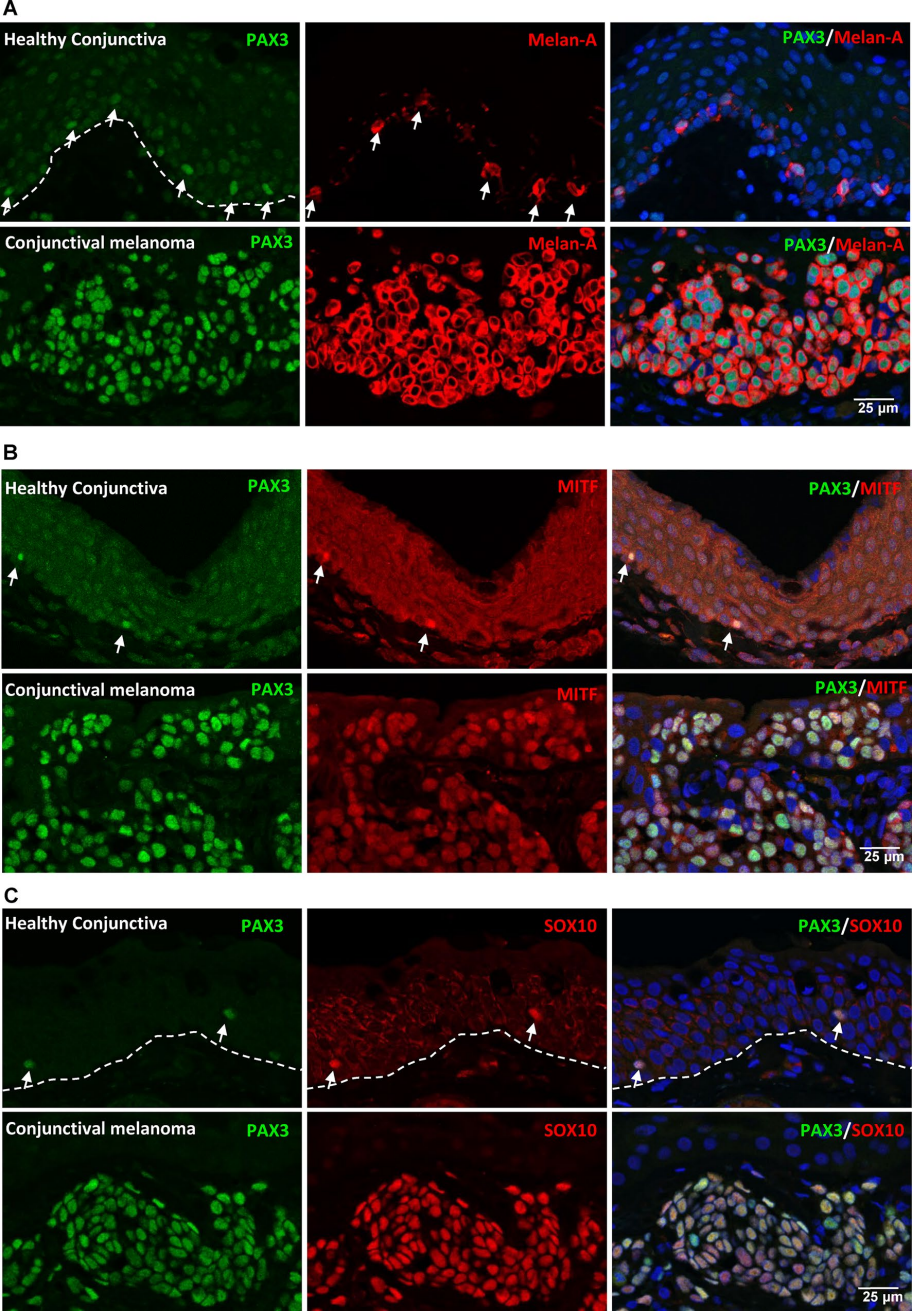
Melanocyte marker expression in conjunctival melanoma. (**A**) Double immunofluorescence staining of conjunctival melanoma paraffin sections showing co-expression of PAX3 (green, arrows) and Melan-A (red). Melan-A staining intensity higher in melanoma tissues compared to healthy tissues. (**B** and **C**) Expression of melanocyte-specific transcription factors in healthy and melanoma tissues: microphthalmia-associated transcription factor (MITF) is expressed in both no notable difference, while SOX10 is expressed in both with strongest staining observed in melanoma tissues.

**Fig. 7:**
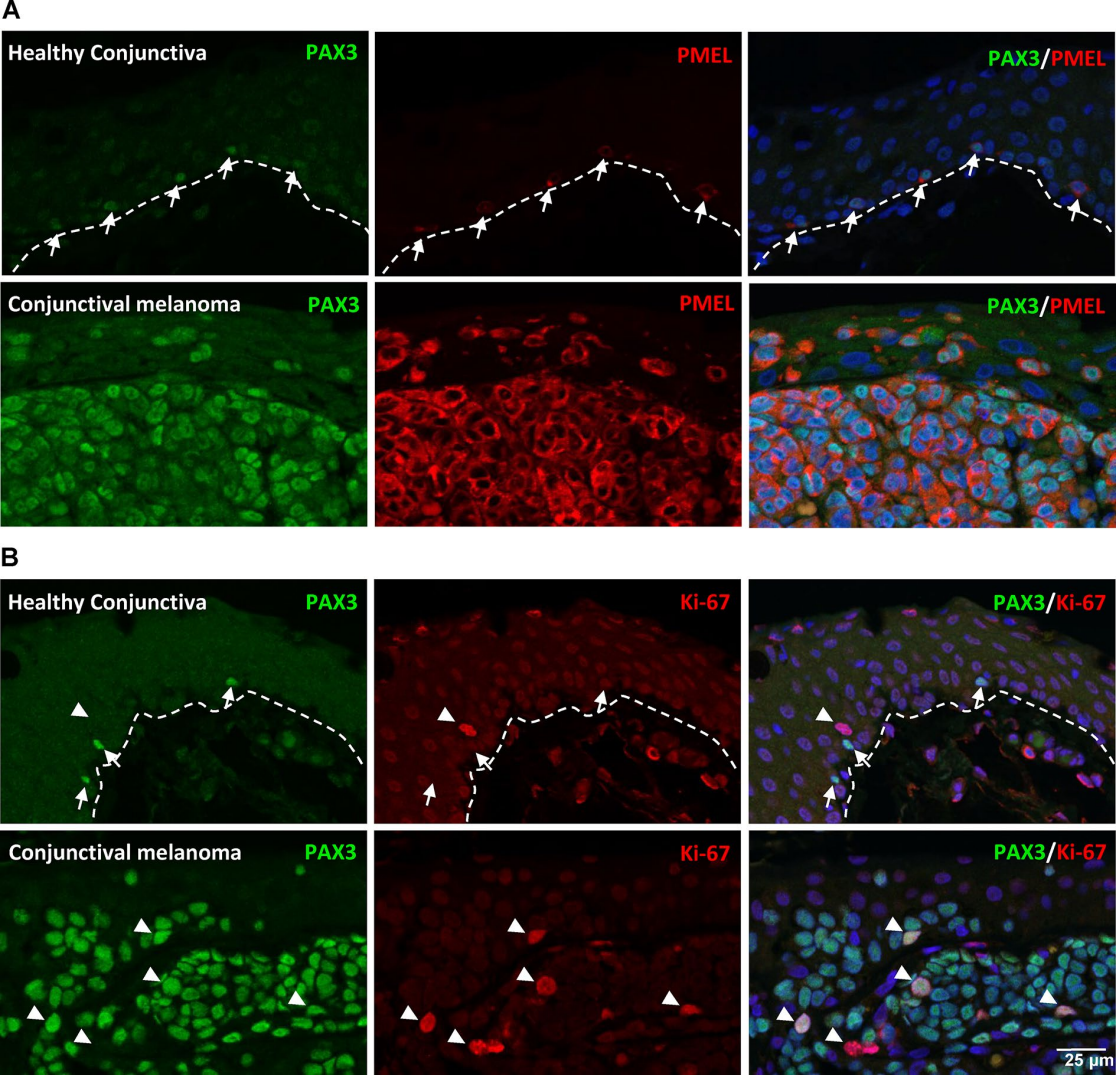
Pigmentation and proliferation markers in conjunctival melanoma. (**A**) Double immunofluorescence staining showing expression of the pigmentation marker HMB45: weakly stained or undetectable in healthy conjunctiva, with strong expression in melanoma tissues. (**B**) Proliferation status of PAX3^+^ cell: in healthy tissues, PAX3^+^ cells do not express the proliferation marker Ki-67 (arrows, red), while in melanoma tissues, PAX3^+^ cells stain positive for Ki-67 (arrowheads), indicating proliferation status.

To investigate the relevance of PAX3 expression in conjunctival melanoma, transcriptional profiling datasets from a previously published study on healthy conjunctiva and conjunctival melanoma were retrieved from the Gene Expression Omnibus (GSE148387). Intriguingly, data analysis revealed significant upregulation of PAX3 in conjunctival melanoma of poor (7.8 ± 2.3-fold, p = 0.01) and good prognosis (18.7 ± 5.5-fold, p = 0.01) compared to healthy conjunctival tissue (Fig. 5A). No significant prognosis-associated difference in normalized readcounts of melanoma samples was observed (Fig. 5A). To further validate these findings, immunohistochemical analysis was performed on paraffin-embedded sections of conjunctival or conjunctival/limbal melanomas. The first patient presented with a pigmented lesion of the temporal conjunctiva since 1995, which had gradually increased in size over the years with focal corneal invasion at 5 o’ clock (Fig. 5B). Histologically epitheloid melanoma cell nests were found subepithelially confirming a confirming a conjunctival/limbal melanoma (HE staining, Fig. 5c). In three other cases the tumour consisted also of epitheloid cells while spindle cells predominated in the remaining two cases (HE staining, Supplementary Fig. 2A). Immunohistochemical analysis revealed strong expression of PAX3 (green) in conjunctival melanoma compared to their respective healthy counterpart tissues (Fig. 5d). This increased PAX3 expression was noted in both the epithelial and stromal components of the melanoma lesions (Fig. 5D). Double immunofluorescence staining of conjunctival melanoma paraffin sections demonstrated that all PAX3^+^ cells (green, arrows) co-expressed the melanocyte marker Melan-A (red), and the intensity of Melan-A staining was notably higher in the melanoma tissues relative to healthy tissues (Fig. 6A). However, the expression of Melan-A at the transcriptional level, as determined by RNA-sequencing data analysis, did not show any significant difference between healthy and melanoma tissues (Suppl.Figure 2B). The melanocyte-specific transcription factors microphthalmia-associated transcription factor (MITF) and SOX10 were expressed in both healthy and melanoma tissues, but no difference was observed for MITF, while strong staining for SOX10 was observed in melanoma tissues (Fig: 6B and 6 C). These findings were consistent with the RNA-sequencing data analysis (Supplementary Fig. 2b). Furthermore, the pigmentation marker HMB45 was weakly stained or undetectable in healthy conjunctiva, whereas strong expression was observed in melanoma tissues (Fig. 7A). Similar expression patterns for HMB45 were observed in the RNA-sequencing data (Supplementary Fig. 2B). Notably, while PAX3^+^ melanocytes in healthy tissues did not express the proliferation marker Ki-67 (arrows, red), the PAX3 + cells within the melanoma tissues stained positive for Ki-67 (Fig. 7b; arrowheads), indicative of their proliferative status. The RNA-sequencing data also showed a significant upregulation of Ki-67 (MKI67) in melanoma tissues compared to healthy tissues (Suppl. Figure 2B).

**Fig. 8:**
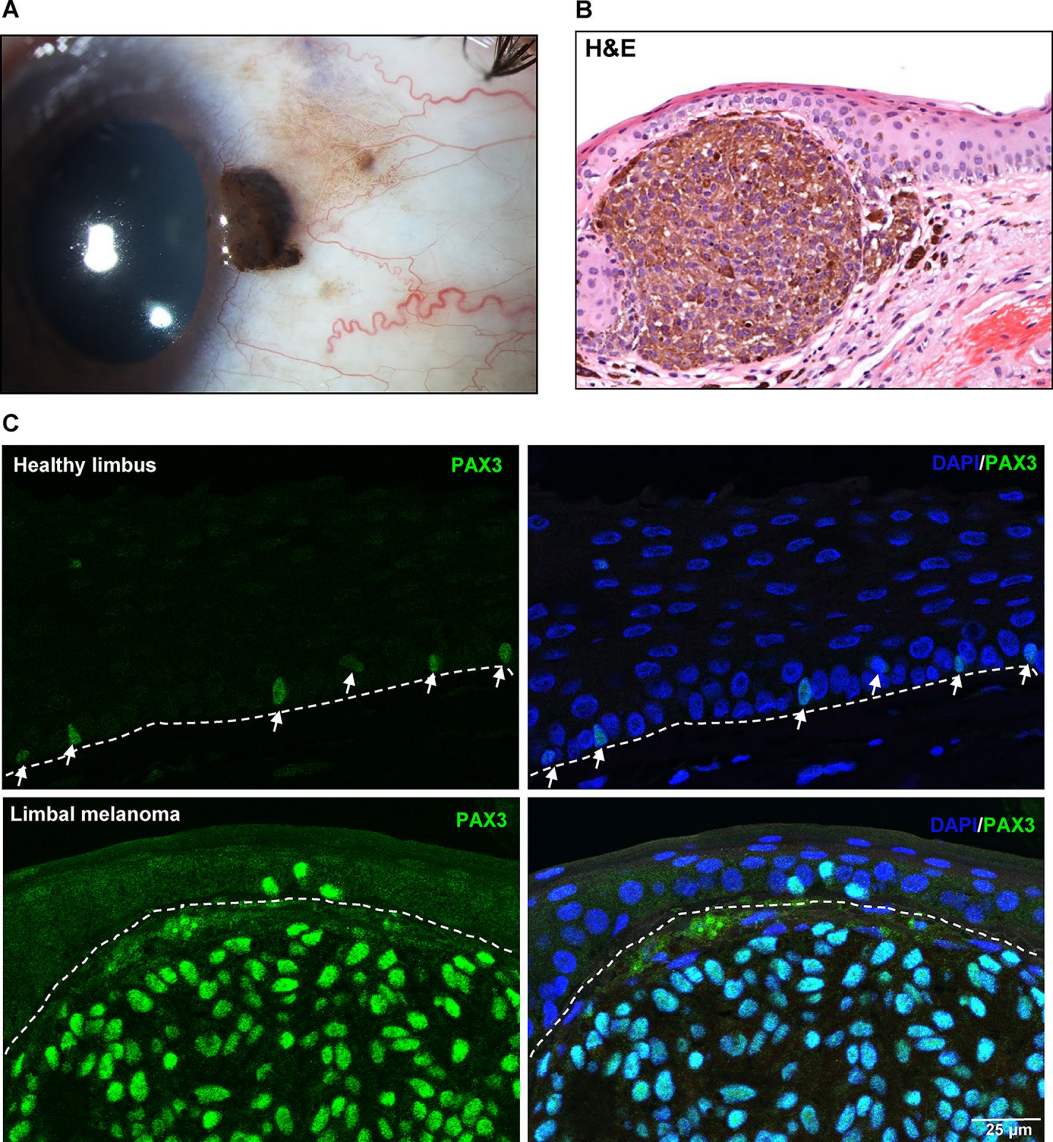
PAX3 expression in limbal melanoma: (**A**) Representative clinical image of limbal melanoma showing a pigmented lesion. (**B**) Representative histological specimen of limbal melanoma characterized by the nodular supraepithelial invasion of heavily pigmented melanoma cells. (**C**) Immunohistochemical analysis revealing the strong expression of PAX3 (green) in limbal melanoma tissues compared to healthy counterpart tissue. Dashed line represents the basement membrane.

In conjunctival/limbal melanomas, one sample exhibited a probable melanocytic origin from the limbus. A patient had a prominent pigmented conjunctival/limbal tumor exhibiting an increasing size over time (Fig. 8a). Upon histological examination utilizing hematoxylin and eosin (H&E) staining, a diagnosis of conjunctival/limbal melanoma was established. This malignancy was characterized by the nodular subepithelial invasion of heavily pigmented melanoma cells, which were covered by an epithelium exhibiting moderate squamous metaplasia (Fig. 8b). Immunohistochemical analysis revealed strong expression of PAX3 (green) in the conjunctival/limbal melanoma tissues (Suppl. Figure 3) compared to their respective healthy counterpart tissues. This increased PAX3 expression was observed in both the epithelial and stromal components of the melanoma lesions (Fig. 8C). The pattern of double immunostaining for PAX3 and other melanocytic markers were similar to that observed in conjunctival melanoma (Suppl. Figure 3).

## Discussion

The study investigated the expression patterns of PAX transcription factors in the limbal stem cell niche, specifically focusing on LEPC, LMSC and LM. The findings from both in vitro and immunohistochemical analysis revealed distinct expression profiles of PAX family members among these cell populations, highlighting their potential roles in regulating stem cell behaviour and lineage commitment, and maintenance of the melanocyte phenotpye. Among the PAX family proteins, PAX3 exhibited predominant expression in LM compared to LMSC and LEPC, aligning with its known function in melanocyte development and pigmentation^28,29^. The predominant expression of PAX3 in LM suggests a role in regulating the melanogeneic program and maintaining the melanocyte phenotype within the limbal stem cell niche. These findings were further supported by the immunocytochemical and Western blot analyses, which confirmed the expression of PAX3 protein in cultured LM. PAX3, a key transcriptional regulator involved in melanocyte development, has also been implicated in melanoma pathogenesis. During melanocytogenesis, PAX3 forms a complex with SOX10 to activate the master regulator MITF, which in turn binds to the DCT (Dopachrome Tautomerase) promoter, allowing melanocyte differentiation^10^. However, PAX3 can also inhibit melanocyte differentiation by competing with MITF for binding to DCT^10^. This dual role suggests PAX3’s involvement in the regulation of melanocyte lineage commitment and differentiation.

The immunohistochemical localization studies corroborated the in vitro findings and provided additional insights into the spatial distribution and cellular identitiy of PAX3-expressing cells within the limbal stem cell niche and adjacent conjunctival tissue. Double immunostaining experiments revealed that PAX3^+^ cells in the limbal and conjunctival tissues co-expressed the melanocyte markers Melan-A (a melanocyte specific protein), and SOX10 (a transcription factor involved in melancyte development), but not epithelial keratin markers (CK12, CK13, CK15), suggesting they represent the population of melanocytes residing within the basal layer of these epithelia. This data indicates that all the ocular surface melanocytes (Melan A^+^/SOX10^+^) in the limbal and conjunctival epithelium express the PAX3 protein. The expression of PAX3 in conjunctival melanocytes is in line with an earlier study where PAX3 expression has been reported by RNA-sequencing and in situ hybridization techniques^30^. Interestingly, the expression pattern of PAX3 in LM was consistent in fresh and organ-cultured limbal tissues, indicating that the culture conditions did not significantly alter the expression of this transcription factor in this specific cell population^31^. This finding provides confidence in the reliability of the cell culture model used in the study and its relevance to the in vivo situation. The absence of PAX3 expression in vimentin^+^ stromal cells suggests that PAX3 may not play a significant role in regulating the stromal cell population within the ocular surface epithelial microenvironment under the experimental conditions tested. The PAX2/5/8 gene subfamily and PAX6 play an important role in the ocular development, maintenance and pathologic processes^32,33^. In the ocular suface, PAX6 expression patterns are known to be dynamic during development and vary across different stages of cellular differentiation^16,34–36^. In this context, our previous study addressed discrepancies in the literature regarding PAX6 expression in adult limbal cells^16,35–37^, demonstrating the absence of PAX6 in LM and LMSC through complementary approaches, including immunohistochemical localization on fresh and organ-cultured corneas and protein level analysis via Western blotting with two antibody clones^17^. The current study builds upon these findings, further confirming the absence of PAX6 expression in LMSC and LM at both tissue and molecular levels. While some earlier reports suggested PAX6 expression in certain limbal cell populations (limbal niche cells) under specific conditions^36,38^, these discrepancies might be attributed to several factors including differences in cell purity^39^, isolation and culture techniques^36,38^, different cell populations tested i.e. limbal niche cells versus LMSC^17,36^, a paucity of investigations on immunohistochemistry in fresh human corneoscleral tissues^36,39^, and different imaging modalities used for detection. The complex nature of PAX6 expression during development and cellular differentiation warrants further investigation to fully understand it’s temporal and spatial regulation in the limbal stem cell niche. PAX8 expression has been reported in normal ocular tissues, including the lens epithelium, iris pigment epithelium, and ciliary body epithelium and in iris and ciliary body-derived neoplasms^40^. The relatively low expression levels of PAX4, PAX8, and PAX9, with no significant differences among the cell types, suggest that these proteins may not play a major regulatory role in the limbal stem cell niche. Additionally, the undetectable expression of PAX1, PAX2, PAX5, and PAX7 indicates that these proteins are likely not crucial for the maintenance and function of the limbal stem cell niche in this context. The combined in vitro and immunohistochemcial localization data highlight differential PAX gene family expression in the limbal stem cell niche, with PAX6 predominant in LEPC and PAX3 in LM, suggesting crucial roles in maintaining their respective stem cell populations and regulating differentiation trajectories. PAX3’s predominance in ocular surface melanocytes indicates potential involvement in regulating the melanogenic program, pigmentation, and stem cell properties within ocular surface regions. Future studies should investigate how PAX3 regulates gene expression and signaling pathways underlying stem cell maintenance, proliferation, lineage commitment, and melanocyte development in the limbal niche as well as the conjuntival tissues.

The other key novel findings from this study are the significant upregulation of the transcription factor PAX3 in conjunctival melanoma tissues compared to healthy conjunctival tissue and the co-upregulation of PAX3 with melanocyte markers such as Melan-A, human melanoma black (HMB45), SOX10 and the proliferation marker Ki-67 in the melanoma cells. These results imply a potential role for PAX3 in the initiation, development, and progression of conjunctival melanoma. In the present study, analysis of previously published RNA sequencing data from conjunctival melanoma and healthy conjunctiva samples^24,41^ revealed a significant upregulation of PAX3 in melanoma tissues, irrespective of prognosis. However, since these datasets were derived from bulk tissue samples, the observed PAX3 upregulation could be attributed to either an increased number of melanocytes within the tumor or enhanced PAX3 expression within the melanoma cells themselves. To elucidate this, we performed immunohistochemical analysis of conjunctival melanoma tissues as well as in limbal melanoma tissues, which confirmed strong PAX3 expression in melanoma cells compared to melanocytes in healthy counterpart tissues. Several studies have reported PAX3 expression in various stages of melanoma progression, including primary tumors as well as advanced stages^42,43^. Its expression has also been utilized as a staging marker and for detecting circulating melanoma cells^44,45^. These findings, along with observed PAX3 expression in healthy melanocytes, suggest that PAX3 dysregulation may contribute significantly to melanoma development^46^. However, further molecular validation, tumor marker assesments and histological analyses in both primary and metastatic melanomas with varying prognoses are needed to fully elucidate its role.

Our study implemented a double immunofluorescence staining approach on paraffin sections utilizing combinations of the melanocytic markers Melan-A and MITF with the proliferation markers Ki-67 and pigmented melanocytic marker HMB45, enabling the sensitive and specific identification of cells exhibiting both melanocytic and proliferative characteristics, which is crucial for better diagnosing (pre)malignant melanocytic lesions. The co-expression of PAX3 with Melan-A, a specific marker for melanocytes, confirms that the PAX3^+^ cells in the melanoma tissues are of melanocytic origin. Interestingly, while Melan-A protein expression was higher in melanoma compared to healthy tissues, its mRNA levels did not differ significantly. This discrepancy could be attributed to the inherent limitations of working with RNA extracted from formalin-fixed, paraffin-embedded tissue sections, which is often degraded and of lower quality compared to RNA from fresh or frozen tissues, leading to inaccuracies in gene expression quantification, especially for transcripts with low abundance or those prone to degradation, as well as potential interference from chemical modifications introduced during the formalin fixation process^47^. The observed upregulation of SOX10, a neural crest transcription factor, in melanoma tissues is in line with its established role in melanocyte specification and maintenance^24,48,49^. PAX3 and SOX10 are known to regulate the expression of melanogenic enzymes, including MITF, which is a master regulator of melanocyte development and melanogenesis^50,51^. However, no significant difference in MITF expression was observed between healthy and melanoma tissues, suggesting that additional factors may be involved in regulating MITF expression in conjunctival melanoma. The increased expression of HMB45, a marker for melanosomal proteins, in melanoma tissues aligns with the expected higher melanogenic activity in melanoma cells compared to normal melanocytes^52^. This finding, combined with the upregulation of PAX3 and SOX10, suggests a potential link between these transcriptional regulators and the enhanced melanogenic phenotype observed in conjunctival melanoma. The co-localization of PAX3 and the proliferation marker Ki-67 in melanoma cells, but not in healthy melanocytes, suggests that PAX3 may be associated with increased proliferative capacity and tumor growth. This finding is supported by previous studies that have linked PAX3 expression to enhanced proliferation and survival of skin melanoma cells^53,54^.

In conclusion, this study provides novel insights into the potential involvement of PAX3 in the development and progression of conjunctival melanoma. The co-expression of PAX3 with melanocyte markers and its association with increased proliferation suggest that PAX3 may contribute significantly to melanoma development. Further investigation into the mechanisms by which PAX3 contributes to conjunctival/limbal melanoma pathogenesis could lead to the identification of potential therapeutic targets and improve our understanding of this rare but one of the most aggressive ocular malignancies.

## Electronic supplementary material

Below is the link to the electronic supplementary material.

Supplementary Material 1

## Data Availability

The datasets used and/or analysed during the current study available from the corresponding author on reasonable request.

## Declarations

### Generative AI in scientific writing

During the preparation of this work the authors used Claude AI in order to improve readability and language. After using this tool, the authors reviewed and edited the content as needed and take full responsibility for the content of the publication.
