# Supplementary material for: PAX3 expression patterns in ocular surface melanocytes
Source: Sci Rep. 2025 Apr 11;15:12472. doi: 10.1038/s41598-025-90318-3 (PMC11992251; doi:10.1038/s41598-025-90318-3)
Supplement: Supplementary file 1 — Supplementary Material 1 [file 41598_2025_90318_MOESM1_ESM.docx]

PAX3 expression patterns in ocular surface melanocytes

Eva Ulrich^1^, Sebastian Kistenmacher^1^, Gottfried Martin^1^, Ursula Schlötzer-Schrehardt^2^, Berthold Seitz^3^, Claudia Auw-Hädrich^1^, Günther Schlunck^1^, Thomas Reinhard^1^, Naresh Polisetti^1^

^1^Eye Center, Medical Center - Faculty of Medicine, University of Freiburg, Killianstrasse 5, 79106, Freiburg, Germany

^2^Department of Ophthalmology, University of Erlangen-Nürnberg, Erlangen, Germany

^3^Department of Ophthalmology, Saarland University Medical Center, Homburg/Saar, Germany

^*^**Corresponding author:** Naresh Polisetti, Eye Center,
Medical Center – University of Freiburg, Killianstrasse 5, 79106 Freiburg Germany.
Phone +49 761 270 40010, fax +49 761 270 40630,
e-mail: [naresh.polisetti@uniklinik-freiburg.de](mailto:naresh.polisetti@uniklinik-freiburg.de)

**Keywords:** Limbal stem cells, Limbal stem cell niche, Limbal niche cells, Mesenchymal stromal stem cells, Melanocytes, Limbal epithelial progenitor cells, Melanoma, Limbal melanoma, Conjunctival melanoma, PAX3, PAX6, Aniridia, Limbal stem cell deficiency

**Supplementary figures and tables:**

**
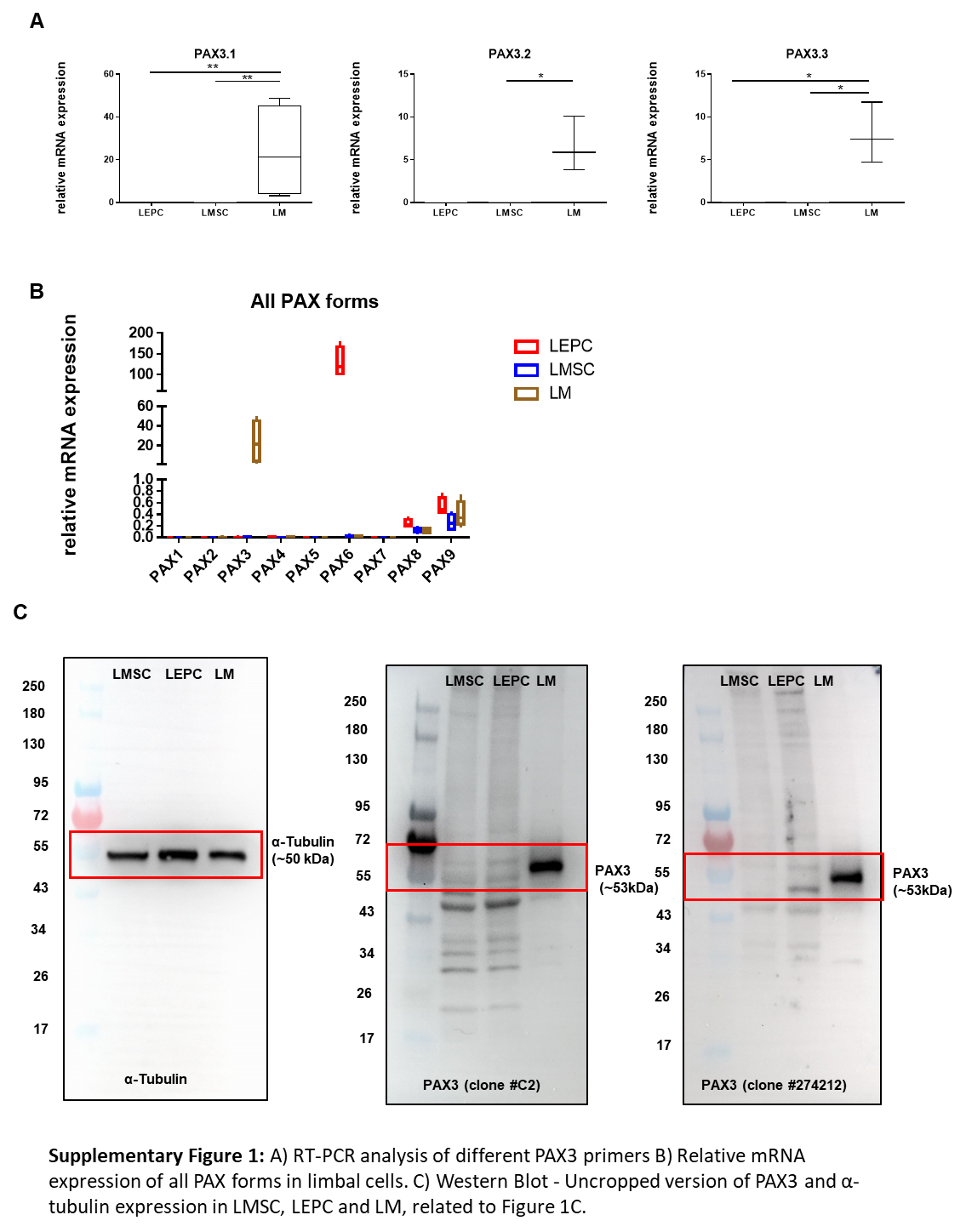
**


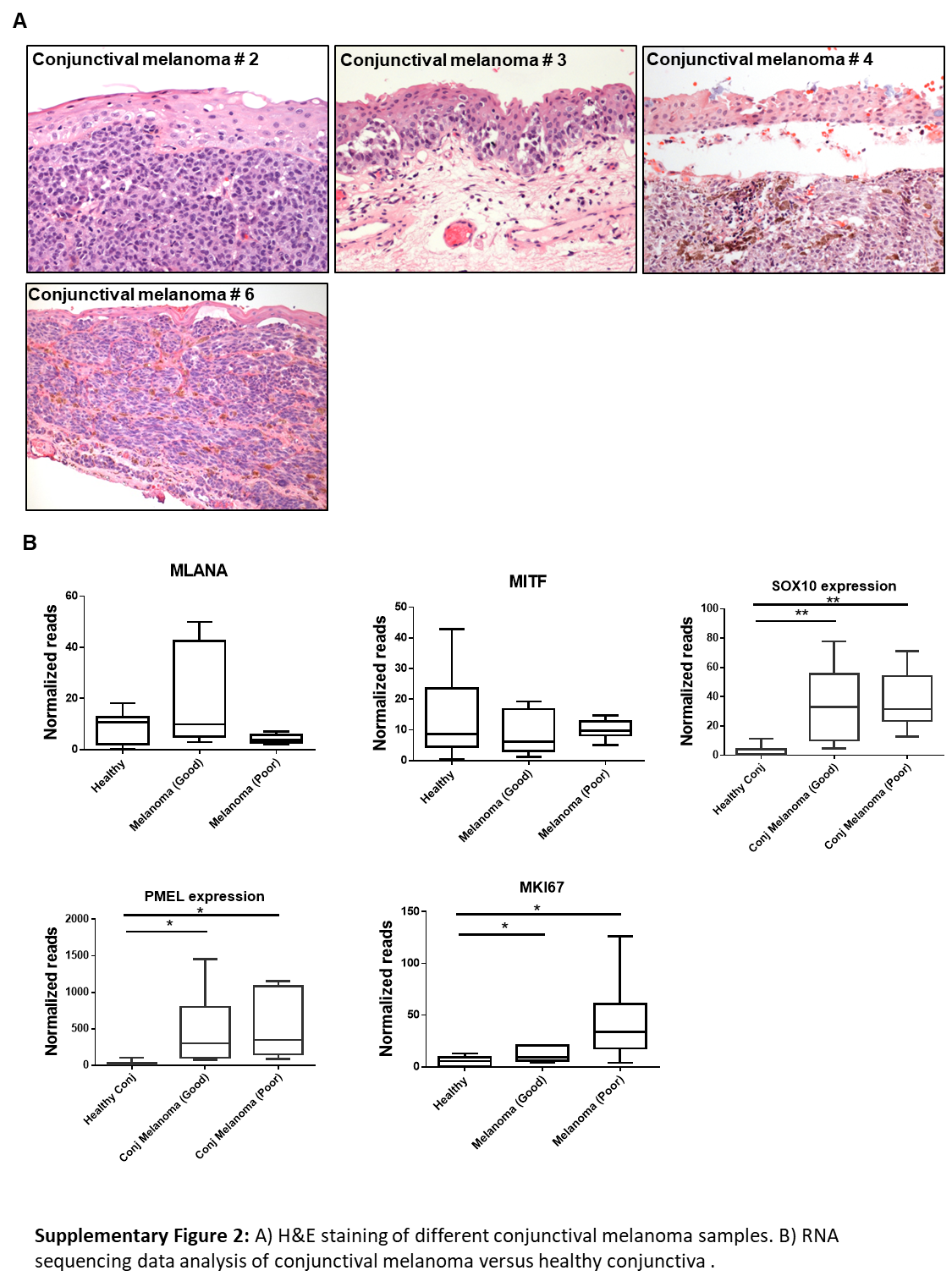


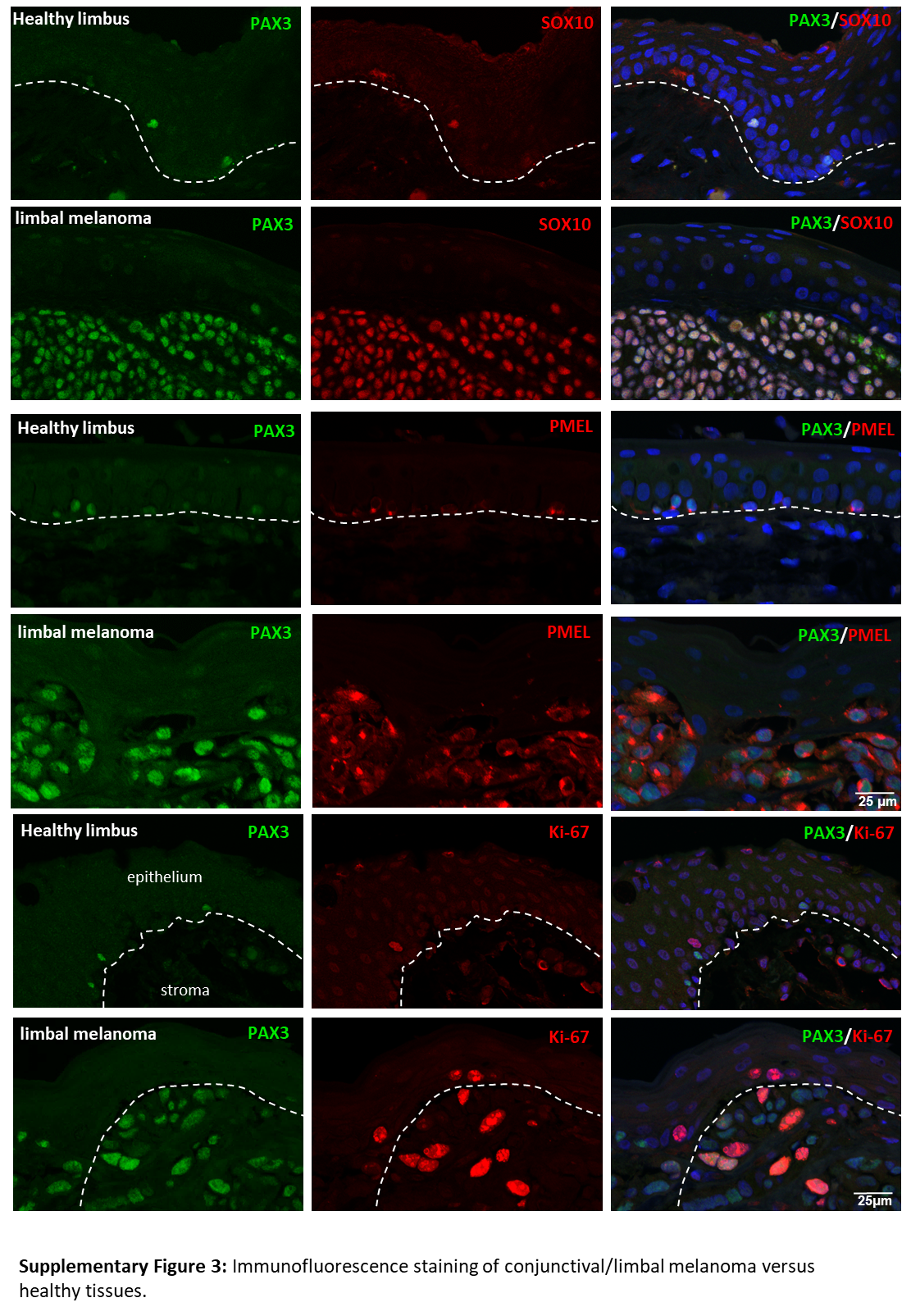


|  | Age [years] | Gender | Clinical findings and history | Other diagnoses |
| --- | --- | --- | --- | --- |
| Conjunctival/  Limbal melanoma | 63 | f | Conjunctival pigmentation since 1995 increasing in size;  Overlap of conjunctival tumor with limbus | Breast carcinoma 2013 treated by chemotherapy and Tamoxifen,  basal cell carcinoma right cheek |
|  | 60 | f | Since 1 year vascularized lesion increasing in size,  clinically conjunctival intraepithelial neoplasia was suspected;  Overlap of conjunctival tumor with the limbus | - |
|  | 67 | m | Fleshy, vascularized tumour | Unknown, external case |
|  | 68 | m | Pigmented conjunctival tumour | Unknown, external case |
|  | 46 | m | Prominent pigmented limbal tumour increasing in size | Schizophrenia |
|  | 56 | f | Pigmented conjunctival/limbal tumour since approx. 6 months | Back problems |

**Supplementary Table 1: Details of melanoma cases used in the study.**

| **Antibody** | **Ref No. #** | **Company** | **Host** | **Dilution ICH/ICC** | **Dilution WB** |
| --- | --- | --- | --- | --- | --- |
| PAX3 | 38-1801 | ThermoFisher Scientific | Rabbit | 1:200 – 1:400 | 1:1000 |
| PAX3 | ab180754 | abcam | Rabbit | 1:50 | 1:1000 |
| PAX3 | HPA063659 | Sigma-Aldrich | Rabbit | 1:200 |  |
| PAX3 | MAB2457 | R&D* | Mouse | 1:100 – 1:200 | 1:500 – 1:1000 |
| PAX3 | 12412S | Cell signalling | Rabbit | 1:100 | 1:1000 |
| PAX3 | AB_528426 | DSHB* | Mouse | 1:200 | 1:1000 – 1:2000 |

**Supplementary Table 2: Details of PAX3 antibodies used in the study. *Only antibodies that performed effectively under the tested working conditions.**

| **Antibody** | **Ref. No. #** | **Company** | **Host** | **Dilution ICH/ICC** | **Dilution WB** |
| --- | --- | --- | --- | --- | --- |
| MelanA | CF506018 | ThermoFisher Scientific | Mouse | 1:400 |  |
| MLANA | ab210546 | abcam | Rabbit | 1:500 |  |
| TRP1 | ab235447 | abcam | Rabbit | 1:1000 |  |
| SOX10 | ab155279 | abcam | Rabbit | 1:500 |  |
| PAX6 | 60433S | Cell Signalling | 60433S | 1:400 |  |
| CK12 | ab185627 | abcam | Rabbit | 1:50 |  |
| CK15 | ab52816 | abcam | Rabbit | 1:100 |  |
| pan-CK | NB600-579 | Novus Biologicals | Rabbit | 1:100 |  |
| Vimentin | 5741S | Cell Signalling | Rabbit | 1:200 |  |
| CK13 | EPR3671 | abcam | Rabbit | 1:200 |  |
| GAPDH | MAB374 | Sigma-Aldrich | Mouse |  | 1:5000 |
| Vinculin | V9131 | Sigma-Aldrich | Mouse |  | 1:400 |
| α-Tubulin | T9026 | Sigma-Aldrich | Mouse |  | 1:6000 |

**Supplementary Table 3: Details of antibodies used in the study.**

| **PAX1** |  |  |  |
| --- | --- | --- | --- |
| PAX1_5_21  PAX1_3_21 | 5‘  5‘ | GGCGAGCATTCACTGAGGAT  CCTCCACACCCCACATGAAA | 3‘  3‘ |
| **PAX2** |  |  |  |
| PAX2_5_14  PAX2_3_14 | 5'  5' | ACAAACGACAGAACCCGACT  ATTGTCACAGATGCCCTCGG | 3‘  3‘ |
| PAX2_5_13  PAX2_3_13 | 5'  5' | CCGTTGTCAAGACACGATGG  CTCGGAGCCCCTCTTTGTTT | 3‘  3‘ |
| **PAX3** |  |  |  |
| PAX3_5_63  PAX3_3_63 | 5'  5' | ATCAGCCGCATCCTGAGAAG  TCCTCTGCCTCCTTCCTCTC | 3‘  3‘ |
| PAX3_5_63  PAX3_3_63 | 5'  5' | GCCGTCAGTGAGTTCCATCA  GGCCTTCTTCTCGCTTTCCT | 3‘  3‘ |
| PAX3_5_3  PAX3_3_3 | 5'  5' | AGACCTCTTACCAGCCCACA  CGTGCTTTGGTGTACAGTGC | 3‘  3‘ |
| **PAX4** |  |  |  |
| PAX4_5_66  PAX4_3_66 | 5'  5' | TTTTAGCAGTCCCCTGGCAG  ACAGCTGATAGCAGGAGGGA | 3‘  3‘ |
| **PAX5** |  |  |  |
| PAX5_5_49  PAX5_3_49 | 5'  5' | TGGCTGTGACAGTATGCAGG  TGATCTGTGTTTCCAGGGGC | 3‘  3‘ |
| **PAX6** |  |  |  |
| PAX6_5_36  PAX6_3_36 | 5'  5' | ACCAATTCCACAACCCACCA  TGTGAGGGCTGTGTCTGTTC | 3‘  3‘ |
| **PAX7** |  |  |  |
| PAX7_5_14  PAX7_3_14 | 5'  5' | ACAAGAGGGAAAACCCAGGC  GTCACAGTGCCCATCCTTCA | 3‘  3‘ |
| **PAX8** |  |  |  |
| PAX8_5_7  PAX8_3_7 | 5'  5' | GACCATCTGATGCCCCAGAG  TGAGGTACCCAGCGTTCAAC | 3‘  3‘ |
| **PAX9** |  |  |  |
| PAX9_5_51  PAX9_3_51 | 5'  5' | CCCCAATTCCCAGGTCTCAC  AGTCCGTACAGCCAGCTTTC | 3‘  3‘ |
| **GAPDH** |  |  |  |
| GAPDH_5_82  GAPDH_3_82 | 5'  5' | CATCCTGGGCTACACTGAGC  GTCAAAGGTGGAGGAGTGGG | 3‘  3‘ |

**Supplementary Table 4: Details of primers used in the study.**
